# Robust EEG-Based Decoding of Auditory Attention With High-RMS-Level Speech Segments in Noisy Conditions

**DOI:** 10.3389/fnhum.2020.557534

**Published:** 2020-10-07

**Authors:** Lei Wang, Ed X. Wu, Fei Chen

**Affiliations:** ^1^Department of Electrical and Electronic Engineering, Southern University of Science and Technology, Shenzhen, China; ^2^Department of Electrical and Electronic Engineering, The University of Hong Kong, Hong Kong, Hong Kong

**Keywords:** EEG, temporal response function (TRF), auditory attention decoding, speech RMS-level segments, signal-to-noise ratio

## Abstract

The attended speech stream can be detected robustly, even in adverse auditory scenarios with auditory attentional modulation, and can be decoded using electroencephalographic (EEG) data. Speech segmentation based on the relative root-mean-square (RMS) intensity can be used to estimate segmental contributions to perception in noisy conditions. High-RMS-level segments contain crucial information for speech perception. Hence, this study aimed to investigate the effect of high-RMS-level speech segments on auditory attention decoding performance under various signal-to-noise ratio (SNR) conditions. Scalp EEG signals were recorded when subjects listened to the attended speech stream in the mixed speech narrated concurrently by two Mandarin speakers. The temporal response function was used to identify the attended speech from EEG responses of tracking to the temporal envelopes of intact speech and high-RMS-level speech segments alone, respectively. Auditory decoding performance was then analyzed under various SNR conditions by comparing EEG correlations to the attended and ignored speech streams. The accuracy of auditory attention decoding based on the temporal envelope with high-RMS-level speech segments was not inferior to that based on the temporal envelope of intact speech. Cortical activity correlated more strongly with attended than with ignored speech under different SNR conditions. These results suggest that EEG recordings corresponding to high-RMS-level speech segments carry crucial information for the identification and tracking of attended speech in the presence of background noise. This study also showed that with the modulation of auditory attention, attended speech can be decoded more robustly from neural activity than from behavioral measures under a wide range of SNR.

## Introduction

The human auditory system enables listeners to follow attended speakers and filter out background noises effortlessly, known as the “cocktail party” effect (Cherry, 1953). The ability of selective attention of target streams from interferences is not only grounded in the acoustic properties of clean and noisy speech (e.g., spatial, spectral, and temporal cues), but also accounts for responses in any part of the central auditory pathway (Snyder et al., 2012). Some researchers have investigated speech signal processing methods via the examination of neural responses to facilitate the attended speech recognition of hearing assistance devices in complex auditory scenes (e.g., Christensen et al., 2018; Miran et al., 2018; Somers et al., 2019). Several advantages could be derived from the incorporation of neural responses in speech signal processing. For instance, the optimal parameters of speech recognition algorithms could be determined by individual neural responses in auditory central pathways (Loeb and Kessler, 1995). Furthermore, as listeners’ intentions could be detected without verbal feedback (Miran et al., 2018), the incorporation of neural feedback into some speech-processing algorithms and its application in hearing prostheses (e.g., hearing aids and cochlear implants) have been considered to be effective approaches for improvement of the hearing ability of listeners with communication impairments (e.g., Mc Laughlin et al., 2012; Aroudi et al., 2019).

Several recent magnetoencephalographic and electroencephalographic (EEG) studies have shown that neural responses during auditory selectivity tasks correlate more strongly with attended than with ignored speech (e.g., Ding and Simon, 2012; Horton et al., 2013; O’Sullivan et al., 2015). Auditory attention decoding models have been established to describe the relationship between continuous speech and ongoing cortical recordings (Alickovic et al., 2019). The linear temporal response function (TRF) model (Crosse et al., 2016) has been used widely to predict EEG responses to speech (i.e., the encoding model; e.g., Di Liberto et al., 2015) and to reconstruct speech from associated EEG signals (i.e., the decoding model; e.g., Ding and Simon, 2012; Mirkovic et al., 2015; Teoh and Lalor, 2019) using off-line regression techniques. Several speech features contribute greatly to the decoding of auditory attentional focus in multi-speaker situations. Given the complex structure of speech, researchers have suggested that the human auditory cortex, together with related brain areas, processes speech using a hierarchical neural structure (Peelle et al., 2010; Ding et al., 2016). Specifically, low-level acoustic cues (e.g., speech spectrograms; Ding and Simon, 2013; Horton et al., 2014) and high-level discrete speech features (e.g., phonemes and semantic context; Di Liberto et al., 2015; Ding et al., 2016; Broderick et al., 2019) show reliable correlations to corresponding neural responses reflected by typical TRF components and neural tracking abilities, indicating that the reconstruction of specific neural representations of attended speech is influenced jointly by different types of speech features under background interference. Among these speech features, amplitude fluctuations of speech stimuli at low frequencies (i.e., the speech temporal envelope) have been used extensively as inputs for the decoding of auditory attention in online daily-life applications (e.g., Mirkovic et al., 2015; Van Eyndhoven et al., 2016; Christensen et al., 2018) employing non-invasive neuroimaging techniques (e.g., EEG). Use of the speech temporal envelope has enabled the achievement of high auditory attention decoding accuracy (e.g., Horton et al., 2014; Kong et al., 2014; Somers et al., 2019), as demonstrated by the reliability of cortical tracking (i.e., neural phase-locking) of attended speech at low brain oscillation frequencies (i.e., the delta and theta bands; Doelling et al., 2014). In envelope-based auditory attention decoding models, however, the cortical tracking of attended speech may be attenuated with decreased speech intelligibility, despite the lack of change in the speech temporal envelope (Ding et al., 2014; Iotzov and Parra, 2019). These findings indicate that the robust neural representation of attended speech is not based simply on speech amplitude modulation, but also depends on crucial information not fully expressed in the speech envelope (Obleser et al., 2012; Ding and Simon, 2014; Drennan and Lalor, 2019). Furthermore, acoustic information inside the speech temporal envelopes could play distinct roles in speech perception (Doelling et al., 2014; Wang et al., 2019). Accordingly, it is important to further explore the neural mechanism operating in such auditory attention models and identify speech cues that are vital for the segregation of attended speech from background noise.

Speech segments carrying distinct intelligibility information may evoke different cortical responses. Broderick et al. (2019) found that speech segments with greater semantic similarity enabled more accurate neural encoding of speech. Di Liberto et al. (2015) showed that distinct phonemic types within continuous speech could be reflected by categorical processing of cortical responses. The speech segmentation method based on the relative root-mean-square (RMS) level has been used extensively for the assessment of segmental contributions to speech intelligibility (e.g., Kates and Arehart, 2005; Ma et al., 2009; Chen and Loizou, 2011, 2012; Chen and Wong, 2013; Guan et al., 2016; Xu et al., 2019; Wang et al., 2019). In the relative RMS-level based segmentation method, the speech was divided into high-RMS-level segments as with threshold level originally proposed in Kates and Arehart (2005), which used the threshold level of 0 dB relative to the overall RMS level of the whole utterance. In this intuitive definition, high-RMS-level regions include those speech segments with RMS level at or above the mean RMS level of the intact utterance, and this definition for high-RMS-level segments was later consistently used in many studies to investigate the perceptual impact of high-RMS-level segments (e.g., Kates and Arehart, 2005; Ma et al., 2009; Chen and Loizou, 2011, 2012; Chen and Wong, 2013; Guan et al., 2016; Wang et al., 2019; Xu et al., 2019), including phonetic constitutions (e.g., Chen and Loizou, 2012; Chen and Wong, 2013; Wang et al., 2019) and contributions to speech intelligibility modeling (e.g., Kates and Arehart, 2005; Ma et al., 2009; Chen and Loizou, 2011; Guan et al., 2016; Xu et al., 2019). Chen and Loizou (2012) compared the impacts of high-RMS-level and high cochlea-scaled entropy to speech intelligibility prediction, and revealed an advantage of high-RMS-level segments against high cochlea-scaled entropy segments to speech intelligibility. Analysis also showed that high-RMS-level segments were dominated with vowels, while middle-RMS-level segments (i.e., from RMS 0 dB to RMS -10 dB) contained more acoustic transitions between vowels and consonants than high-RMS-level segments did (e.g., Chen and Loizou, 2012; Chen and Wong, 2013). The perceptual importance of high-RMS-level segments was demonstrated in several early work on understanding high-RMS-level-only sentences either in quiet or in noise, while the perceptual benefit of high-RMS-level segments to speech perception in noisy conditions was partially attributed to the benefit of large local signal-to-noise (SNR) levels in high-RMS-level segments (e.g., Guan et al., 2016). The aim of the present work was to specially study the performance of high-RMS-level-segment based auditory decoding in noisy conditions; hence, to be consistent with early work (e.g., Kates and Arehart, 2005; Ma et al., 2009; Chen and Loizou, 2011, 2012; Chen and Wong, 2013; Guan et al., 2016; Wang et al., 2019; Xu et al., 2019), this work continued to use 0 dB threshold level to generate high-RMS-level segments, which would provide findings supplementary to our existing knowledge of the impact of high-RMS-level (with 0 dB threshold level) segments on speech perception.

As the relative intensity of background interference may affect the quality of neural tracking of attended speech (Alickovic et al., 2019), the impacts of various SNR conditions on auditory attention decoding in realistic scenarios should also be considered. Generally, low SNRs interfere with attended speech segregation, and the quality of neural tracking of attended speech declines with increasing noise intensity (Kong et al., 2014; Das et al., 2018). In some studies, however, auditory attentional decoding performance remained robust with top-down attentional modulation, regardless of the number of competing speakers or the degree of reverberation (Van Eyndhoven et al., 2016; Aroudi et al., 2019). Reliable neural tracking of attended streams has also been achieved for a range of background noise levels (Ding and Simon, 2013; Fuglsang et al., 2017; Vanthornhout et al., 2019). Hence, the degree of neural tracking that is feasible under various SNR conditions must be understood to enable application of the auditory attentional decoding model to improve the performance of assistive hearing devices in realistic auditory scenarios.

This study was conducted to explore whether auditory attention could be decoded well from high-RMS-level speech segments under various SNR conditions using EEG signals. Moreover, the mechanisms underlying internal neural representations of attended speech were investigated based on the speech temporal envelope, by analyzing speech segments containing crucial intelligibility information (i.e., high-RMS-level segments). First, the two-speaker mixed sentences were presented to subjects at various SNRs during ongoing EEG recording. Then, the TRF was used to describe the relationships between EEG signals and the features of attended and ignored speech (i.e., the temporal envelopes of intact speech and high-RMS-level segments alone) under different SNR conditions (Crosse et al., 2016). Auditory attention decoding performance was assessed by examining correlations between reconstructed and actual speech signals (Alickovic et al., 2019). We hypothesized that: (1) the neural-tracking activities reflected by TRF responses with intact temporal envelopes would be stronger than that with high-RMS-level segments, as the high-RMS-level segments only contain a part of acoustic information; (2) the attention decoding accuracy with high-RMS-level speech segments would not be inferior to that with intact speech in the presence of background interference, as the high-RMS-level segments would carry sufficient information for the segregation and perception of attended speech; and (3) the top-down modulation of auditory attention would facilitate the classification between attended and ignored speech based on the cortical tracking ability, and be insensitive to the change of noise level.

## Materials and Methods

### Participants

Twenty native Mandarin-Chinese listeners (12 men and 8 women) aged 18–27 years participated in this experiment. All subjects reported having normal hearing (pure-tone thresholds < 25 dB at 125–8000 Hz). Written informed consent was obtained from all subjects prior to study participation. The Research Ethics Committee of the Southern University of Science and Technology approved this study.

### Stimuli and Experimental Procedures

The stimuli used in this experiment were four translated short fiction passages written by Maupassant. Two passages (“Boule de Suif” and “In Country”) were narrated by a female Mandarin speaker and two (“My Uncle Jules” and “Les Bijoux”) were narrated by a male Mandarin speaker. The passages were divided into approximately 60-s segments ending with complete sentences, with periods of silence exceeding 0.5 s shortened to 0.5 s to reduce the possibility of attentional switching. The segments were normalized to an equal RMS amplitude. To generate five SNR conditions, the RMS level of the attended stream was fixed, while the ignored stream was either the same or 6 dB, 3 dB stronger/weaker. Mixed speech streams consisted of two fiction passages, one read by the female speaker and the other read by the male speaker. In each trial, the attended stream began 1 s before the ignored stream, and the two streams ended at the same time.

The experiment was conducted in a double-walled sound-shielded room. All stimuli with a sampling rate of 16,000 Hz were presented bilaterally using E-prime 2 (Schneider et al., 2002) via Sennheiser HD 250 headphones at 65 dB SPL. Each subject was asked to sit in a comfortable chair and look at a fixation point on a computer screen in front of him/her. A total of 100 trials without repetitions of auditory stimuli was presented to each subject under five SNR conditions (6, 3, 0, −3, and −6 dB). The stories were presented in their correct order trial by trial. Each block consisted of five trials under the same SNR condition, followed by an attended speech–related four-multiple-choice question (responses were made by button press). Prior to each block, an on-screen reminder directed the subject to pay attention to the female or male stream. The subjects were given approximately 3-min breaks after every two blocks. During each break, the experimenter explained the main idea of the auditory stimuli stated in the former block to subjects to ensure that the previous story content not affected the understanding of stimuli in the next block. Each SNR condition consisted of four blocks across subjects and condition presentation was randomized. Behavioral performance was recorded as the percentage of correctly answered questions within the same SNR condition for each subject.

### EEG Data Recording

Sixty-four-channel scalp EEG signals were recorded using a SynAmps RT amplifier (NeuroScan, Charlotte, NC, United States) with sampling at 500 Hz. Scalp electrodes were placed following the extended international 10/20 system, with two additional electrodes placed at the left and right mastoids (Homan et al., 1987). A reference electrode was placed at the nose tip, and two electrooculography (EOG) electrodes were placed above and below the left eye, respectively. All channel impedances were kept below 5 kΩ. The participants were asked to minimize body movements to avoid motion artifacts.

### Data Analysis

#### EEG Signal Preprocessing

Offline EEG signal preprocessing was performed with the EEGLAB toolbox (Delorme and Makeig, 2004; Mognon et al., 2011) using Matlab 2015b (MathWorks Inc., Natick, MA, United States). EEG waveforms were re-referenced with the averaged waveforms from the left and right mastoids. Fourth-order Butterworth filtering was conducted with a passband of 1–50 Hz in both forward and backward directions to eliminate phase shifts. The filtered data were segmented into epochs, and each epoch was set to contain the whole length of a trial and a second pre-stimulus baseline. These EEG epochs excluded the first second of each EEG recording after the onset of mixed speech to reduce the effects of neural onset responses. Typical artifacts (i.e., eye blinks, heartbeats, and EOG components) were removed using independent component analysis, via selection using the ADJUST algorithm (Mognon et al., 2011) and labeling by visual inspection in each subject. On average, six independent components (standard deviations = 2) were deleted across subjects, and the remaining components were projected back into channel space for further analyses. In order to decrease subsequent processing time, all EEG data were down-sampled to 100 Hz. Subsequently, as the low-frequency neural responses were phase-locked to speech envelopes (Di Liberto et al., 2015; O’Sullivan et al., 2015), the continuous EEG data were digitally filtered with three kinds of band-pass filters (i.e., 2–8, 8–15, and 15–30 Hz) by a zero-phase finite impulse response filter and the filter order was determined as the three times the ratio of the sampling frequency to the lower-cutoff frequency (Shamma et al., 2011; Mai et al., 2016).

#### Speech Temporal Envelopes Extraction

**FIGURE 1:**
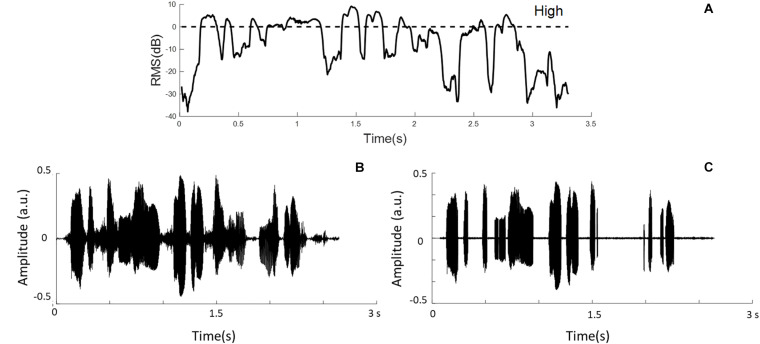
**(A)** Example segments of relative root-mean-square (RMS) energy representations. The dashed line shows the boundaries of the high-RMS-level region. **(B)** The waveform of the original sentence. **(C)** The sentence containing only high-RMS-level segments.

Speech temporal envelopes that represented amplitude fluctuations and high-RMS-level speech segments were used in this study. Hamming windows with the block size of 16 ms and 50% overlap between adjacent windows were used to divide sentences into short-term segments. Then, the signal intensity of each windowed segment was calculated and classified based on the relative RMS level. High-RMS-level segments were at and above the RMS level of each whole utterance (i.e., >0 dB), as defined previously (Kates and Arehart, 2005; Kewley-Port et al., 2007; Chen and Loizou, 2011). The duration of the high-RMS-level segments accounts for 31.83% of the intact speech stimuli in this experiment. Figure 1 shows the relative RMS-level intensity and the defined boundary of the high-RMS-level segments. Furthermore, an example sentence and its high-RMS-level segments are displayed in Figure 1. Each temporal envelope was computed by taking the absolute values of the Hilbert transform from intact stimuli and high-RMS-level segments. These envelopes were down-sampled to the same sampling rate of the EEG signals (i.e., 100 Hz), and then filtered digitally at three frequency bands (i.e., 2–8, 8–15, and 15–30 Hz) using a 150th order zero-phase finite impulse-response band-pass filter. Subsequently, speech temporal envelopes were generated corresponding to the attended and ignored streams at three frequency bands, respectively.

#### TRF Estimation and EEG Prediction

**FIGURE 2:**
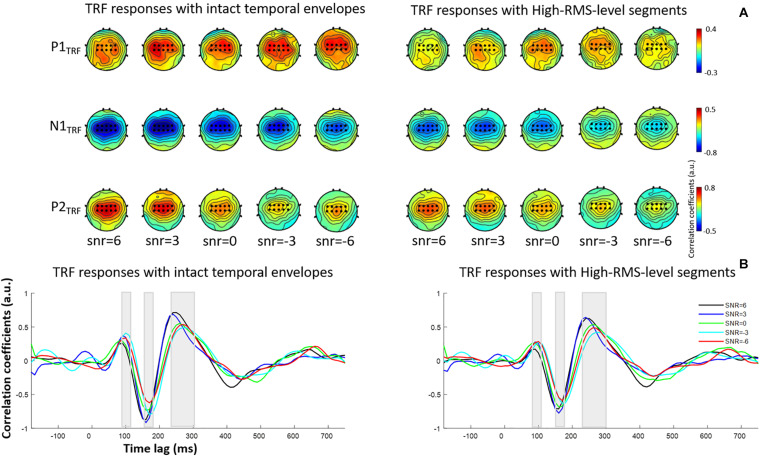
**(A)** Topological distributions of TRF responses at P1_TRF_, N1_TRF_, and P2_TRF_ components with intact temporal envelopes (left) and high-RMS-level segments (right). The electrodes marked as black dots are used to conduct further analyses. **(B)** Grand-averaged estimated temporal response function (TRF) responses with intact temporal amplitude envelopes (left) and high-RMS-level-only envelopes (right) in five SNR conditions.

**FIGURE 3:**
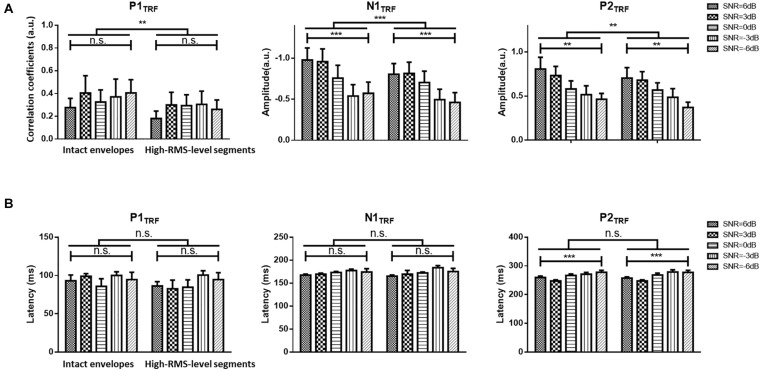
**(A)** Statistical results (mean ± standard deviation) for TRF values with intact and high-RMS-level-only envelopes at three typical deflections across subjects. **(B)** Statistical results (mean ± standard deviation) for TRF latencies with intact and high-RMS-level-only envelopes at three typical deflections across subjects in five SNR conditions. ***P < 0.001, **P < 0.01 (prediction difference). n.s., no significant difference (analysis of variance). SNR, signal-to-noise ratio.

Temporal response functions were used to explain how the cortical responses encoded speech stimuli (e.g., Ding and Simon, 2012; Di Liberto et al., 2015; Broderick et al., 2019). TRF responses were regarded as a filter that described the linear mapping between the temporal envelopes of stimuli and neural responses in this study. The TRF responses were analyzed to describe the relationship between the target speech and corresponding EEG signals. The magnitude and latency of TRF responses were sensitive to the stimulus amplitude, i.e., the increased TRF magnitude and the decreased TRF latency were shown with the augment of stimulus amplitude (e.g., Drennan and Lalor, 2019). The TRF responses affected by the SNR level and the speech temporal envelope other than the stimulus amplitude were mainly investigated in this study. In this study, the amplitudes of target speech were normalized across SNR conditions, while the SNR levels of the mixed stimuli were generated by different amplitudes of the ignored speech. Additionally, the speech envelopes extracted from the intact speech and high-RMS-level segments were normalized before the TRF calculations. The amplitude-normalized envelopes between the intact speech and high-RMS-level segments reduced the effects of stimulus amplitude on TRF responses. TRFs estimation was performed using the mTRF toolbox (Crosse et al., 2016). The encoding model for stimuli and corresponding neural responses is represented by the following equation:

(1) $$r\,\left(t,\,n\right)=\sum_{\tau }w\,\left(\tau ,n\right)\,s\,\left(t-\tau \right)+\varepsilon \,\left(t,n\right),$$

where the TRF – *w*(τ,*n*) – models the transformation for a specified range of time lags, τ, relative to the stimulus feature, *s*(*t*), at the instantaneous time; *s*(*t*) represents the speech envelope at each sampled time; *r*(*t*, *n*) is the EEG response at channel *n*; and ε(*t*,*n*) is the residual response at each channel. The *w*(τ,*n*) was given by minimizing the mean-squared error (MSE) between the actual and predicted EEG responses. The TRF calculated window τ was first conducted from −200 to 800 ms and corresponding TRF responses were further presented in Figure 2 at fronto-central electrodes. Ridge regression was used to select the appropriate regularization parameter for TRF estimation. The optimal ridge regression parameter was determined using a leave-one-out cross-validation approach, wherein every trial was decoded by the averaged decoder parameters trained on the other trials across conditions and subjects. The regularization parameter for TRF estimate was varied for the range of 2^0^, 2^2^, …, 2^12^, and the optimal value of 2^8^ was determined for all stimulus conditions, which led to the highest correlation between the actual neural responses and those predicted by auditory stimuli. Subsequently, the TRF estimations were constructed from the temporal envelopes of intact speech and speech containing only high-RMS-level segments under different SNR conditions, respectively. Previous studies revealed that the TRF responses with a range of time lag yielded similar components as those in the event-related potentials (e.g., Lalor et al., 2009; Kong et al., 2014). The correlation coefficient between the speech envelope and the corresponding neural response was presented as the TRF value in each time lag. The polarity of TRF responses indicated the relationship between the direction of cortical current (i.e., negative or positive) and the decrease or increase of envelope power. The positive TRF responses reflected that a positive cortical voltage tracked the speech envelope power increase; and similarly, a negative voltage on scalp responded to the speech envelope power decrease (Kong et al., 2014). According to the scalp topographies of the typical TRF components, the amplitude and latency were further analyzed at the fronto-central channels (i.e., the black dots in Figure 2A). The TRF amplitudes and latencies of three typical deflections (i.e., P1_TRF_, N1_TRF_, and P2_TRF_) were further analyzed across subjects at the specific statistical window (i.e., 80 ∼ 110, 150 ∼ 180, and 230 ∼ 300 ms) for each component (see Figure 2B). Figure 3 illustrates the mean and standard deviations of TRF responses in these typical components across subjects.

#### Stimulus Reconstruction and Attended Speech Identification

A decoding model was generated to estimate different speech features from ongoing neural responses at various SNRs. Similar to TRF calculation, the decoder was established by considering the stimulus–response system as a linear, time-invariant system (Mirkovic et al., 2015). In the following equation, the decoder – *g*(τ,*n*) – represents linear mapping from the neural response, *r*(*t*, *n*), to the stimulus feature, *s*(*t*):

(2) $$\hat{s}\,\left(t\right)=\sum_{n}\sum_{\tau }r\,\left(t+\tau ,n\right)\,g\,\left(\tau ,n\right),$$

where $\hat{s}\,\left(t\right)$ represents the reconstructed stimulus envelope, *n* is the EEG channel, and τ indicates a specified range of time lags. The time-lag window between 0 and 400 ms was used in the decoding model, as this time range reflected the most information in TRF responses. Ridge regression was used to avoid overfitting. A leave-one-out cross-validation approach was used to optimize the regularization parameter by maximizing the accuracy of speech reconstruction, and the MSE was conducted across trials to avoid overfitting and get the optimal ridge value. Furthermore, this defined decoding model was used to assess correlations between reconstructed and actual temporal envelopes of attended and ignored speech. When the reconstructed speech envelope correlated more strongly to attended than to ignored speech, the attended speech in a mixed speech sample was considered to be identified correctly based on decoding from EEG signals. The auditory attention decoding accuracy with different SNRs was assessed according to the percentage of correct identifications among trials from subjects. Except for calculating the auditory attention decoding accuracy with the whole trial duration (i.e., 60 s), the effects of decoding window durations were also analyzed by calculating the decoding accuracy with shorter epoch durations (e.g., 30, 10, and 2 s).

### Statistical Analyses

Statistical analyses were performed with SPSS 20.0 (SPSS Inc., Chicago, IL, United States) using repeated-measures analysis of variance (ANOVA). First, two-way ANOVA was used to examine the effects of speech features, SNR conditions, and their interaction on TRF responses. Pearson correlation values were calculated to assess the relationships between reconstructed and actual speech features of attended and ignored speech. Attended speech decoding performance across subjects was analyzed using two-way ANOVA with the two main factors of different speech features and SNRs. Finally, topological decoding weights in the left and right hemispheres of the temporal cortex under different SNR conditions were assessed using two-way ANOVA. The distribution of these samples was not significantly different from a normal distribution using the Shapiro–Wilk test (all *P* > 0.05). The Mauchly’s test was used to assess whether these hypotheses were adequate for the assumptions for repeated-measures ANOVA. ANOVAs generated *F* scores and *P*-values, and *post hoc* model comparisons were performed using Bonferroni-corrected paired *t*-tests. The effects of SNRs on the accuracy of subjects’ responses to questions about attended speech were examined using the non-parametric Kruskal–Wallis test.

## Results

### TRF Estimation and Neural Responses of Speech

Figure 2 displays TRF responses to attended speech with the intact and high-RMS-level–only temporal envelopes under the various SNR conditions in the delta and theta bands. The TRFs contained typical deflections, one negative and two positive (P1_TRF_, N1_TRF_, and P2_TRF_), reflecting robust neural tracking of attended speech in the presence of background interference. The topological distributions across the statistical windows of the three components show the strongest responses in fronto-central regions (see Figure 2A). Figure 2B illustrates the averaged TRF responses of electrodes located at the fronto-central position across subjects in each condition. Speech features had a main effect, with the intact-speech temporal envelopes having higher TRF correlation coefficients than the high-RMS-level–only envelopes at all three deflections [P1_TRF_: *F*(1,19) = 8.641, *P* = 0.008, $\eta_{\text{p}}^{2}$ = 0.313; N1_TRF_: *F*(1,19) = 33.354, *P* < 0.001, $\eta_{\text{p}}^{2}$ = 0.637; P2_TRF_: *F*(1,19) = 11.274, *P* = 0.003, $\eta_{\text{p}}^{2}$ = 0.372]. A main effect of the SNR was also detected at N1_TRF_ [*F*(4,76) = 3.765, *P* = 0.008, $\eta_{\text{p}}^{2}$ = 0.165] and P2_TRF_ [*F*(4,76) = 4.019, *P* = 0.005, $\eta_{\text{p}}^{2}$ = 0.175]. No significant interaction effect was found between these two main factors in TRF correlation coefficients of all components [P1_TRF_: *F*(4,76) = 0.390, *P* = 0.390, $\eta_{\text{p}}^{2}$ = 0.052; N1_TRF_: *F*(4,76) = 1.463, *P* = 0.222, $\eta_{\text{p}}^{2}$ = 0.071; P2_TRF_: *F*(4,76) = 0.830, *p* = 0.510, $\eta_{\text{p}}^{2}$ = 0.042]. The shorter of TRF lag is found with the higher SNR in the P2_TRF_ component [*F*(4,76) = 7.567, *P* < 0.001, $\eta_{\text{p}}^{2}$ = 0.296]. Figure 3 illustrates the detailed TRF correlation coefficients and time lags across subjects in each condition.

### Neural Reconstruction of Speech and Auditory Attention Decoding Accuracy

**FIGURE 4:**
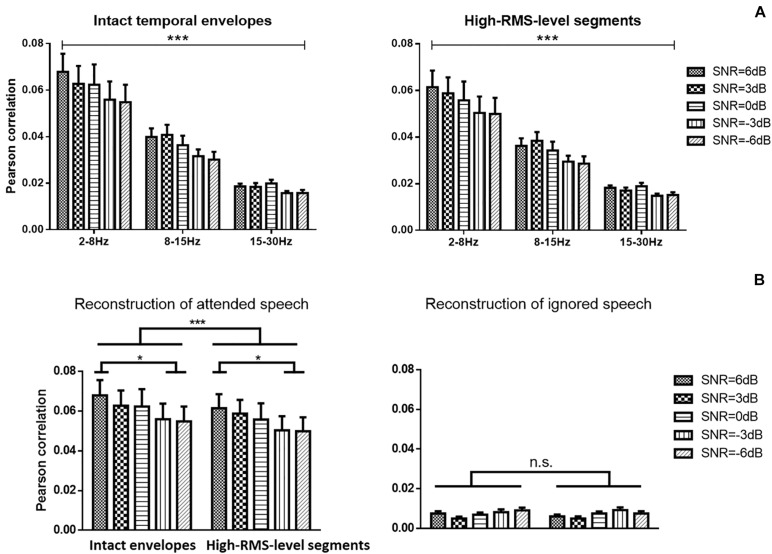
**(A)** Speech envelope prediction correlations under various signal-to-noise ratio (SNR) conditions with the band-pass filter at 2–8, 8–15, and 15–30 Hz. Error bars display mean ± standard deviation. **(B)** Speech envelope prediction correlations under various signal-to-noise ratio (SNR) conditions for attended and ignored streams at the 2–8 Hz frequency band. *P < 0.05, ***P < 0.001 (prediction differences). n.s., no significant difference (analysis of variance).

Figure 4 displays the average Pearson correlation coefficients between estimated and original envelopes of attended speech in different frequency bands and the error bars present the standard deviations across subjects. A three-way repeated ANOVA analysis was conducted to reveal the speech reconstruction performance affected by the speech temporal envelope, SNR level, and frequency band. There was no significant interaction effect across these three factors [*F*(8,152) = 1.790, *P* = 0.083, $\eta_{\text{p}}^{2}$ = 0.090]. There was a significant main effect of the speech temporal envelopes on correlations between estimated and actual speech [*F*(1,19) = 34.885, *P* < 0.001, $\eta_{\text{p}}^{2}$ = 0.660], indicating a significantly higher decoding performance with the intact temporal envelopes than with high-RMS-level segments. A significant decline in the strength of neural tracking of attended speech across SNRs was detected [*F*(4,76) = 7.685, *P* < 0.001, $\eta_{\text{p}}^{2}$ = 0.299]. The Bonferroni adjustment for multiple comparisons revealed that the speech reconstruction performance at 6 dB SNR was higher than −3 dB (*P* = 0.002) and −6 dB (*P* = 0.004) SNR. The frequency bands showed significant main effects on the reconstruction accuracy of the attended speech [*F*(2,38) = 36.124, *P* < 0.001, $\eta_{\text{p}}^{2}$ = 0.667]. The low-frequency band from 2 to 8 Hz showed the higher Pearson correlation compared to the frequency bands at 8–15 Hz and at 15–30 Hz (all *P* < 0.001 with multiple pairwise comparisons by Bonferroni correction). Therefore, the subsequent analyses were carried out with the frequency band at 2–8 Hz. Figure 4B shows the correlations between reconstructed and actual attended and ignored speech in the low-frequency band from 2 to 8 Hz. A two-way repeated ANOVA was analyzed to measure the effects of speech temporal envelope and SNR level. Correlations between reconstructed and actual speech were weaker for high-RMS-level speech segments than for intact speech [*F*(1,19) = 34.014, *p* < 0.001, $\eta_{\text{p}}^{2}$ = 0.642]. A significant decline in the strength of neural tracking of attended speech across SNRs was detected [*F*(4,76) = 5.251, *p* = 0.001, $\eta_{\text{p}}^{2}$ = 0.217], with Bonferroni-corrected pairwise comparison revealing significant differences at −3 dB (*p* = 0.037) and −6 dB (*p* = 0.034) relative to 6 dB. No significant interaction effect between speech features and SNR was observed for the attended speech [*F*(4,76) = 2.043, *p* = 0.097, $\eta_{\text{p}}^{2}$ = 0.097] or ignored speech [*F*(4,76) = 1.665, *P* = 0.168, $\eta_{\text{p}}^{2}$ = 0.085] decoding model with the frequency band at 2–8 Hz.

**FIGURE 5:**
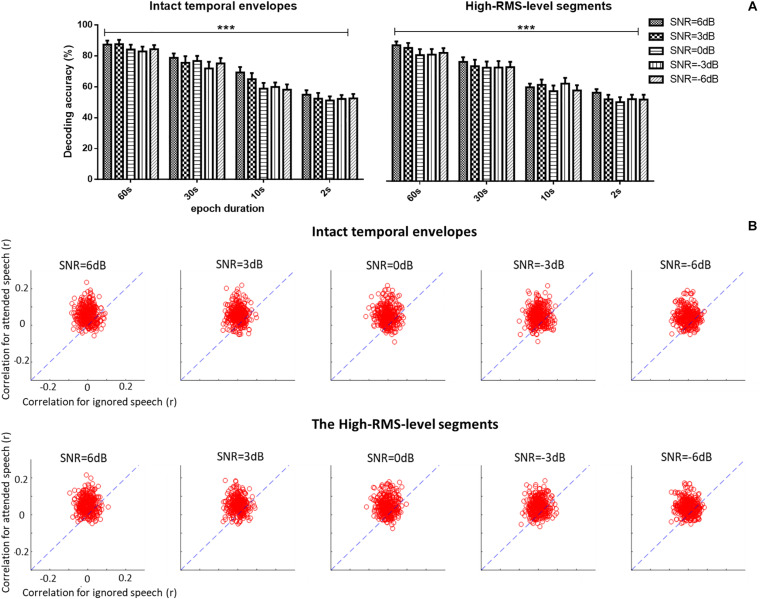
**(A)** Average auditory attention-decoding accuracy across subjects with the intact temporal envelopes and high-RMS-level segments for each signal-to-noise ratio (SNR) with the duration of decoding window at 60, 30, 10, and 2 s. Error bars display mean ± standard deviation. ***P < 0.001. n.s., no significant difference (analysis of variance). **(B)** Scatter plots of the correlation of attended vs. ignored streams across all trials and subjects for each signal-to-noise ratio (SNR) with 60 s decoding window length. Points above the blue dashed lines indicate the correct identification of attended speech.

A three-way repeated ANOVA analysis was carried out to examine the effects of three factors (i.e., the epoch duration of attention decoding, speech temporal envelope, and SNR level) and their interactions on the auditory attention decoding performance based on corresponding EEG signals (see Figure 5A). No significant interaction effect among the three factors was observed [*F*(12,228) = 0.795, *P* = 0.655, $\eta_{\text{p}}^{2}$ = 0.040]. Different SNR levels had no significant effects on the auditory attention decoding performances [*F*(4,76) = 1.288, *P* = 0.282, $\eta_{\text{p}}^{2}$ = 0.063]. A significant decrease of the correlations between the reconstructed and actual speech was shown with the shorter epoch duration of decoding window [*F*(3,57) = 78.637, *P* < 0.001, $\eta_{\text{p}}^{2}$ = 0.805]. The intact and high-RMS-level based speech temporal envelopes showed distinct effects on auditory attention decoding performance with different epoch durations of decoding window. Decoding accuracy performances declined with high-RMS-level segments compared to those with intact temporal envelopes at the 10 s decoding duration [*F*(1,19) = 5.270, *P* = 0.033, $\eta_{\text{p}}^{2}$ = 0.217], while no significant differences were displayed between the intact and high-RMS-level segments with the epoch decoding duration at 60 s [*F*(1,19) = 4.394, *P* = 0.051, $\eta_{\text{p}}^{2}$ = 0.188], 30 s [*F*(1,19) = 1.802, *P* = 0.311, $\eta_{\text{p}}^{2}$ = 0.054] and 2 s [*F*(1,19) = 0.054, *P* = 0.819, $\eta_{\text{p}}^{2}$ = 0.003]. With these results on the effect of epoch duration, the further analyses in this study were processed with the whole length of epoch (i.e., 60 s) because it showed the highest value of reconstruction correlation compared to those with the decoding duration at 30, 10, and 2 s. Figure 5B shows decoding correlations between the reconstructed- and actual-speech temporal envelopes for attended and ignored speech in all trials with the whole duration (e.g., 60 s) of each trail. The dashed lines indicated the decoding performance was equivalent for both speech types.

### Behavioral Performance

**FIGURE 6:**
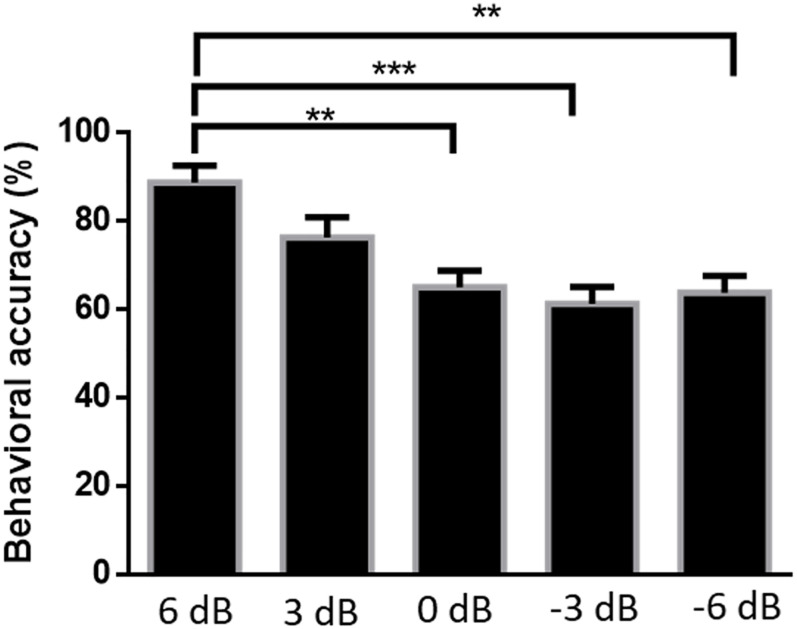
Average percentages of correct responses to questions related to the content of attended speech at each signal-to-noise ratio. Error bars display mean ± standard deviation **P < 0.01, ***P < 0.001 (prediction differences).

Figure 6 shows the accuracy of subjects’ responses to questions related to attended speech under the five SNR conditions. Mean accuracies were 88.75% [standard error of the mean (SEM) = 3.84%] at 6 dB, 76.25% (*SEM* = 4.62%) at 3 dB, 65.00% (*SEM* = 3.80%) at 0 dB, 61.25% (*SEM* = 3.84%) at −3 dB, and 63.75% (*SEM* = 3.84%) at −6 dB. The Kruskal–Wallis test was implemented to compare the accuracy rates of the answered questions in different SNR conditions. The behavioral performance was significantly affected by SNR levels [*H*(4) = 24.574, *P* < 0.001]. There was a strong evidence showing that the behavioral score for 6 dB SNR condition was significantly higher than those for the 0 dB (*P* = 0.004), −3 dB (*P* < 0.001), and −6 dB (*P* = 0.001) SNR conditions, adjusted using the Bonferroni correction. Although the accuracy of attended speaker identification exceeds the random degree of accuracy (i.e., 25%) among all SNR conditions in this behavioral test, the behavioral performance of attended speech accuracy shows a significant decrease with the increased intensity of the competing speaker.

## Discussion

The current study investigated whether high-RMS-level speech segments carried sufficient information for the decoding of auditory attention when speech signals from two talkers were presented concurrently. It also explored the interference of the SNR with the neural tracking of the speech temporal envelope and the cortical selectivity of attended speech. The results showed that: (1) the neural tracking activities to intact and high-RMS-level segments have the same characteristics in topological and morphological distributions, and the TRF responses with high-RMS-level segments showed only weaker magnitudes than that with intact speech envelopes; (2) the speech temporal envelope of high-RMS-level segments could be used to decode auditory attention reliably, with no significant difference in the strength of cortical selectivity from the temporal envelope of intact speech; (3) lower SNRs were associated with worse neural tracking of speech, whereas the accuracy of attended speech selection was insensitive to the level of background noise.

### Contributions of High-RMS-Level Speech Segments to the Auditory Decoding Model

Previous studies have indicated that the EEG-based identification of attended speech in noisy environments depends on robust representation in the temporal cortex of the attended temporal envelope with attention modulation (Van Eyndhoven et al., 2016; Wang et al., 2019). However, the underlying effect of the speech envelope on the decoding of auditory attention remained controversial. As many speech features, such as temporal pitch contours and spatial cues, correlate strongly with the temporal envelope (Shamma et al., 2011), cortical entrainment to attended speech does not depend simply on speech amplitude fluctuations (Obleser et al., 2012; Peelle et al., 2012; Peelle, 2018a). Furthermore, speech segment stimuli of different intensities have been shown to result in distinct morphologies of the stimulus–cortical response model (e.g., the TRF) (Drennan and Lalor, 2019; Wang et al., 2019). Hence, the temporal envelopes of different segments of intact speech likely make diverse contributions to the neural tracking of attended speech in complex auditory scenarios. Speech intelligibility also plays an important role in auditory attention decoding (Iotzov and Parra, 2019), and differs according to the RMS level (Kates and Arehart, 2005; Chen and Loizou, 2011); high-RMS-level segments contain crucial speech intelligibility information (especially in Mandarin), due partially to the large proportions of vowels and tonal information that they contain (Kewley-Port et al., 2007). In this study, the speech envelope for high-RMS-level segments was extracted and utilized as a speech feature to model the neural tracking ability and attended speech selectivity in noisy conditions. Neural tracking activities reflected by TRF amplitudes were worse for high-RMS-level segments than for the intact temporal envelope, indicating that each segment of the speech temporal envelope contributes to the cortical representation of attended speech. These results are consistent with previous reports of interaction effects among distinct speech segments (e.g., prediction strategies and semantic context) in the perception of continuous speech (Golumbic et al., 2012; Ding and Simon, 2013). In this study, the reliably topological and morphological distributions of TRF responses between the intact and high-RMS-level segments suggest that the high-RMS-level segments carry the sufficient acoustic information for neural tracking of the amplitude envelope of auditory stimuli. In addition, we found no difference in the strength of neural selectivity for attended speech between high-RMS-level segments and the intact speech, yielding similar auditory attention–decoding accuracy. This phenomenon could be used to support that high-RMS-level segments contain the high speech intelligibility content, even in noisy conditions (e.g., Chen and Wong, 2013; Guan et al., 2016). However, the contribution of high-RMS-level segments to auditory attention decoding performance could also be affected by the length of the decoding window. In line with previous studies (e.g., Zink et al., 2017), the accuracy of auditory attention detection declined with a shorter duration of the decoding window. In this study, with a shorter duration of the decoding window (e.g., 10 s), more robust detection accuracies of auditory attention decoding were shown with the intact temporal envelopes than those with high-RMS level segments. This suggested that acoustic cues located in other speech segments (e.g., speech onsets and speech silences) could potentially play a vital role in auditory attention detection with a shorter decoding window. In addition, the cortical representation of attended speech does not depend merely on the speech envelope; it is associated with an analysis-by-synthesis process that yields an object-level representation (Ding and Simon, 2012; Golumbic et al., 2012). Therefore, high-RMS-level speech segments, with amplitude fluctuations (i.e., the speech temporal envelope) and containing crucial speech intelligibility information, could be vital for auditory attentional modulation to separate attended speech from background noise (Ding and Simon, 2012; Iotzov and Parra, 2019). These results further suggested the impact of high-RMS-level segments on speech perception in the presence of a competing speaker, as well as the ability to perform auditory attention detection using only certain crucial speech segments. This study mainly used the robust correlations between the speech amplitude fluctuations and corresponding EEG signals to decode auditory attention under noisy conditions. A higher reconstruction accuracy between EEG responses and the speech envelopes was found in the delta and theta bands than that in the higher frequency bands, which was consistent with the literature (e.g., Ding and Simon, 2013; Di Liberto et al., 2015). However, speech features in the time and spectral domain could all affect the speech perception and corresponding cortical responses (e.g., Biesmans et al., 2016; Teng et al., 2019). Future studies could systematically analyze how cortical responses track the speech features at different auditory-inspired narrow bands to better simulate the processing in the auditory peripheral and central systems. Furthermore, this study used the original definition of high-RMS-level segments as those speech segments with RMS level at or above the mean RMS level of the intact utterance (i.e., 0 dB relative to the RMS level of the whole utterance) (Kates and Arehart, 2005), and this threshold level has been consistently used in many studies (e.g., Kates and Arehart, 2005; Ma et al., 2009; Chen and Loizou, 2011, 2012; Chen and Wong, 2013; Guan et al., 2016; Wang et al., 2019; Xu et al., 2019). Only a limited number of work focused on the effect of change in RMS-level threshold on speech perception in the RMS-level based segments (e.g., Chen and Wong, 2013). In future studies, the investigation of different RMS-level thresholds for speech segmentation would be of importance to understand the perceptual contributions in each speech segment. Furthermore, the current study mainly focused on the perceptual contribution of speech segments with high RMS levels, and it warrants further investigations to study the contributions of the other related and important acoustic features (e.g., local SNR and cochlea scaled entropy).

### Auditory Attention Decoding Performance Under Various SNR Conditions

The temporal profiles of the TRFs at the fronto-central positions showed three reliable components for the analysis of neural tracking of attended speech under various SNR conditions in this study. These three typical peaks of TRF responses reflect different neurophysiological processing stages, and are also discovered in previous studies (e.g., Crosse et al., 2016). The P1_TRF_ response remained stable with varying intensity of background noise, and may be related only to the acoustic features of the attended stream (Ding and Simon, 2013; Petersen et al., 2016). The N1_TRF_ and P2_TRF_ components reflect the perception of attended speech separately from background noise (Ding and Simon, 2012, 2014). Their amplitudes declined with the increasing level of competing speech and the P2_TRF_ latencies were significantly prolonged with the lower SNR; together with the observed weaker neural tracking at higher competing-speech intensities, these findings confirm that the intensity of background noise affects the neural tracking of attended speech. In contrast, the SNR between 6 and −6 dB had no significant effect on the tracking of ignored streams in this study. Hence, this study illustrated that SNR levels have different impacts on neural responses to attended and ignored speech, in line with previous findings suggesting that attended and ignored speech are processed independently for the identification of target auditory objects in complex auditory scenarios (Simon, 2015). In addition, auditory attention could lead to the distinct modulation of attended and ignored speech (i.e., enhancement and suppression, respectively) to facilitate the detection of attended auditory objects (Horton et al., 2013; O’Sullivan et al., 2015).

Regardless of the effect of the SNR (e.g., from 6 dB to −6dB) on the neural tracking of attended speech, the auditory attention decoding accuracy was robust (>80%) in this study. This finding further verifies that contributions of auditory attentional modulation to neural selectivity for attended speech (Obleser and Kayser, 2019). Additionally, the behavioral results showed that the attended speech was intelligible, in other words that it could be separated from background noise and understood. This intelligibility may have led to the robust cortical selectivity observed under all SNR conditions in this study. The present work suggested that auditory attention decoding performance with high-RMS-level segments was relatively robust under noisy conditions within a range of SNR levels (i.e., from 6 to −6 dB). This finding provided new evidence on the robust performance in human listeners’ auditory attention decoding task in noisy conditions, and also has important insights to our knowledge on the mechanism of robust EEG-based auditory attention decoding. Further work could focus on how the robust EEG-based auditory attention decoding improves speech perception in challenging listening conditions, e.g., with severe SNR levels (i.e., less than −6 dB), and in cocktail party problems. Moreover, this study illustrated only the effect of the SNR on decoding accuracy for attended speech; other studies have indicated that the degree of the speaker’s position separation (e.g., Dai and Shinn-Cunningham, 2016; Das et al., 2018) and the speaker’s gender and speaking rate (e.g., Peelle, 2018b), jointly influence auditory attention detection with different background noise levels. Listener characteristics (e.g., age and degree of hearing impairment) have also been found to affect individual EEG-based detection (e.g., Dai et al., 2018). Hence, further studies should incorporate consideration of these factors into exploration of the effect of background noise and improvement of the auditory attention detection algorithm with neural feedback in realistic scenarios.

## Conclusion

The present study investigated the contributions of high-RMS-level segments of Mandarin sentences to the EEG-based decoding of auditory attention in the presence of various intensities of competing speech. Although the TRF responses and the activities of neural tracking were decreased for these speech segments, the strength of neural selectivity was comparable to that for intact speech. Similarly, the decoding accuracy of attended speech was robust based on neural activities, and insensitive to the SNR range between 6 dB to −6dB tested in this study. These results suggest that high-RMS-level speech segments are critical for the construction of object-level neural representations of attended speech under various SNR conditions. The study results also indicate that EEG signals can be used to robustly identify the attended speaker when the intensity of the interfering speech increases within a certain range.

## Data Availability Statement

The raw data supporting the conclusions of this article will be made available by the authors, without undue reservation, to any qualified researcher.

## Ethics Statement

The studies involving human participants were reviewed and approved by The Research Ethics Committee of the Southern University of Science and Technology. The patients/participants provided their written informed consent to participate in this study.

## Author Contributions

LW contributed to the design and implement of the experiments, the analysis and interpretation of data, and the writing of the manuscript. EW contributed to the revision of the manuscript and final approval of the submitted version. FC contributed to the design of experiments, the interpretation of data, the revision of the manuscript, and final approval of the submitted version. All authors contributed to the article and approved the submitted version.

## Conflict of Interest

The authors declare that the research was conducted in the absence of any commercial or financial relationships that could be construed as a potential conflict of interest.
